# First-principles investigation of the micromechanical properties of fcc-hcp polymorphic high-entropy alloys

**DOI:** 10.1038/s41598-018-29588-z

**Published:** 2018-07-25

**Authors:** Xiaoqing Li, Douglas L. Irving, Levente Vitos

**Affiliations:** 1 Department of Materials Science and Engineering, KTH-Royal Institute of Technology, 10044 Stockholm, Sweden; 2 Department of Materials Science and Engineering, North Carolina State University, Raleigh, North Carolina 27695 USA; 3 Research Institute for Solid State Physics and Optics, Wigner Research Center for Physics, P.O. Box 49, Budapest, H-1525 Hungary; 4 Department of Physics and Astronomy, Division of Materials Theory, Uppsala University, Box 516, SE-75120 Uppsala, Sweden

## Abstract

High-entropy alloys offer a promising alternative in several high-technology applications concerning functional, safety and health aspects. Many of these new alloys compete with traditional structural materials in terms of mechanical characteristics. Understanding and controlling their properties are of the outmost importance in order to find the best single- or multiphase solutions for specific uses. Here, we employ first-principles alloy theory to address the micro-mechanical properties of five polymorphic high-entropy alloys in their face-centered cubic (fcc) and hexagonal close-packed (hcp) phases. Using the calculated elastic parameters, we analyze the mechanical stability, elastic anisotropy, and reveal a strong correlation between the polycrystalline moduli and the average valence electron concentration. We investigate the ideal shear strength of two selected alloys under shear loading and show that the hcp phase possesses more than two times larger intrinsic strength than that of the fcc phase. The derived half-width of the dislocation core predicts a smaller Peierls barrier in the fcc phase confirming its increased ductility compared to the hcp one. The present theoretical findings explain a series of important observations made on dual-phase alloys and provide an atomic-level knowledge for an intelligent design of further high-entropy materials.

## Introduction

There has been a notable increase of interest in the high-entropy alloys (HEAs) in the last years due to their outstanding properties^1–12^, e.g., excellent strength and fracture toughness, high hardness, good wear and corrosion resistance, and good thermal conductivity. The HEAs contain several elements in equimolar or near equimolar ratios that often possess different ground state crystal structures. However, the majority of these reported alloys exhibit two simple structures, i.e., face-centered cubic (fcc) or body-centered cubic (bcc), whereas only few single-phase HEAs with hexagonal close-packed (hcp) structure have been produced^13–16^. Among those, the hcp HEAs containing rare earth elements did not show compositional segregation, e.g., DyGdHoTbY, but they typically lacked pronounced solid-solution hardening due to the similar size and electronic structure of the constituents. Attempts to synthesize single-phase hcp HEAs without rare earth elements have been rare until now^16^ owing to compositional segregation^17^.

Recently, Li *et al*.^6,12^ developed a new class of HEAs, which exhibits an fcc and hcp phase mixture, such as Cr_10_Mn_30_Fe_50_Co_10_. These so-called dual phase HEAs display a combination of high strength and good ductility opposite to the single-phase fcc HEAs with similar components. Lately, an fcc to hcp phase transformation of Cr_20_Mn_20_Fe_20_Co_20_Ni_20_ and Cr_25_Fe_25_Co_25_Ni_25_ under pressure was reported^18–20^. Remarkably, the hcp phase was retained following decompression to ambient pressure, possibly opening a new, fruitful route for producing HEAs in the hcp structure. Furthermore, finite temperature *ab initio* calculations suggested that the free energy of hcp Cr_25_Fe_25_Co_25_Ni_25_ and Cr_20_Mn_20_Fe_20_Co_20_Ni_20_ is lower than that of fcc at room temperature and below^21,22^.

Mechanical properties of engineering materials are of primary interest because they determine the ability to withstand loads without failure. To further develop high-performance HEAs, it is fundamentally interesting to explore the mechanical properties of these polymorphic HEAs in both the fcc and hcp phases. However, difficulties in preparing pure phase samples have largely prevented the experimental characterization of the individual phases in dual phase HEAs hitherto, and a comparison of the mechanical properties of the two phases is still lacking. Here, using first-principles simulations, we compare for the first time the elastic properties and intrinsic shear strength of five polymorphic HEAs, namely, Cr_25_Fe_25_Co_25_Ni_25_, Cr_20_Mn_20_Fe_20_Co_20_Ni_20_, Cr_20_Mn_20_Fe_30_Co_20_Ni_10_, Cr_20_Mn_20_Fe_34_Co_20_Ni_6_, and Cr_10_Mn_30_Fe_50_Co_10_, in their fcc and hcp phases. These five 3*d* transition metal HEAs were successfully synthesized, and their magnetic properties were characterized^6,12,23,24^. Using the obtained shear strength, we lend fundamental insight into the half-width of the dislocation core that is involved in studying the plastic deformation behavior of these materials.

## Results and Discussion

### Lattice parameters

Table 1 lists the calculated equilibrium lattice parameters as well as available theoretical and experimental data^18–20,25^. For the fcc phase, we can see that our values are very similar to the presented theoretical data calculated by the Vienna ab initio simulation package (VASP) using special quasi-random structures^25^. Both theoretical results are in good agreement with the experimental values^20,25^. For the hcp phase, our data agree well with the available experimental data^18–20^.

**Table 1:** Computed lattice parameters (in Å) as well as available theoretical and experimental data^18–20,25^ of HEAs in the hcp and fcc phases.

HEA	fcc			hcp			
	*a*_EMTO_	*a*_VASP_	*a*_Expt_.	*a*_EMTO_	*a*_Expt._	*c*_EMTO_	*c*_Expt._
Cr_10_Mn_30_Fe_50_Co_10_	3.503	…	…	2.478	…	3.948	…
Cr_20_Mn_20_Fe_34_Co_20_Ni_6_	3.517	…	…	2.487	…	3.979	…
Cr_20_Mn_20_Fe_30_Co_20_Ni_10_	3.519	…	…	2.488	…	3.985	…
Cr_20_Mn_20_Fe_20_Co_20_Ni_20_	3.529	3.540^25^	3.597^25^	2.493	2.544(1)^18^	4.005	4.142(3)^18^
			3.597^18^		2.535(2)^19^		4.138(1)^19^
Cr_25_Fe_25_Co_25_Ni_25_	3.529	3.540^25^	3.575^25^	2.497	2.522^20^	4.025	4.118^20^
			3.574^20^				

### Elastic properties

For a cubic crystal, there are three independent single-crystal elastic constants, i.e., *C*_11_, *C*_12_, and *C*_44_. However, for a hexagonal solid, this number increases to five, namely *C*_11_, *C*_12_, *C*_13_, *C*_33_, and *C*_44_. In addition, *C*_66_ = (*C*_11_−*C*_12_)/2 holds. The obtained elastic constants for the five considered HEAs in their fcc and hcp phases are presented in Table 2. We find that both the fcc and hcp phases of all considered HEAs are elastically stable at zero temperature and pressure judged from the Born criteria^26,27^. The elastic stability is consistent with their experimental observations at zero pressure and room temperature^6,12,18,19^. Moreover, it was found that our obtained elastic constants for Cr_25_Fe_25_Co_25_Ni_25_ and Cr_20_Mn_20_Fe_20_Co_20_Ni_20_ in their fcc phase agree well with recently reported theoretical data^28^.

**Table 2:** Single-crystal elastic constants (in GPa) of HEAs in the hcp and fcc phases.

HEA	fcc			hcp				
	*C*_11_	*C*_12_	*C*_44_	*C*_11_	*C*_12_	*C*_13_	*C*_33_	*C*_44_
Cr_10_Mn_30_Fe_50_Co_10_	258.4	137.6	205.0	484.5	215.0	132.2	573.8	142.3
Cr_20_Mn_20_Fe_34_Co_20_Ni_6_	255.3	152.5	190.6	429.9	197.5	110.9	498.1	117.5
Cr_20_Mn_20_Fe_30_Co_20_Ni_10_	250.0	150.5	186.6	415.9	190.3	104.6	478.7	111.6
Cr_20_Mn_20_Fe_20_Co_20_Ni_20_	240.0	146.9	179.3	375.6	179.1	90.1	433.8	96.8
Cr_25_Fe_25_Co_25_Ni_25_	268.2	175.9	175.7	348.6	182.1	93.1	412.5	81.9

Using the obtained elastic constants, we calculated the single-crystal Young’s modulus *E* in several high-symmetry directions and determined its anisotropy characterized by the anisotropy factor *f*_*E*_ defined in refs^29,30^. *f*_*E*_ can be obtained from the following equations,

1 $${f}_{E}^{{\rm{hcp}}}=\frac{{C}_{33}{C}_{11}-{C}_{13}^{2}}{{C}_{11}^{2}-{C}_{12}^{2}},$$

2 $${f}_{E}^{{\rm{fcc}}}=\frac{2{C}_{44}({C}_{11}+2{C}_{12})}{{C}_{11}({C}_{11}+{C}_{12})-2{C}_{12}^{2}}.$$

A larger deviation of *f*_*E*_ from 1 indicates a larger dependence of *E* on the loading direction.

**Figure 1:**
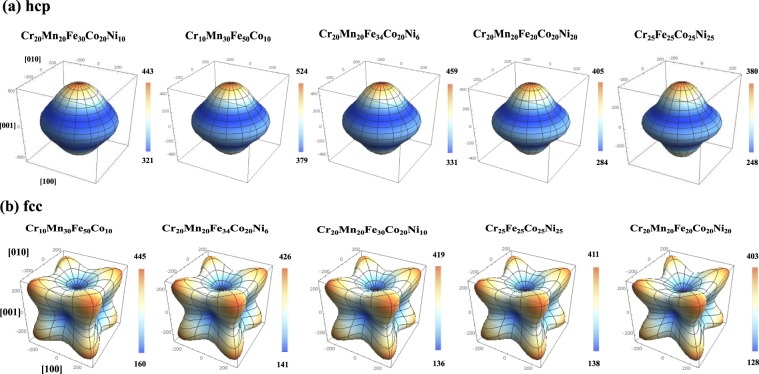
The directional dependence of Young’s modulus *E* (in GPa) of HEAs in the hcp and fcc structures in the order of increasing *f*_*E*_. The Cartesian axes specify the projection of *E* onto the [100], [010], and [001] crystallographic axes. For the hcp phase, the [001] direction of the plot is parallel to the [0001] direction of the hcp unit cell.

For the fcc phase, we can see from Table 3 that the [111] and [001] directions possess the largest and smallest *E*, respectively. From the listed ${f}_{E}^{{\rm{fcc}}}$, it is evident that *E* of Cr_20_Mn_20_Fe_20_Co_20_Ni_20_ is the most anisotropic, whereas Cr_10_Mn_30_Fe_50_Co_10_ is the most isotropic alloy. For the hcp phase, *E* along the [0001] direction is larger than that along the $\mathrm{[2}\bar{1}\bar{1}0]$ direction, and the largest ${f}_{E}^{{\rm{hcp}}}$ is found in Cr_25_Fe_25_Co_25_Ni_25_. The full directional dependence of *E* = *E*([*hkl*]) for all HEAs is shown in Fig. 1 for the hcp and fcc phases, in order of increasing *f*_*E*_ for the sake of comparison. The Young’s modulus surface for the hcp phase is axially symmetric owing to the symmetry of the elastic constant tensor. Furthermore, we can see that there is a correlation between the magnitude of the anisotropy factor *f*_*E*_ and the shape of the *E*([*hkl*]) surface, e.g., hcp Cr_25_Fe_25_Co_25_Ni_25_ and fcc Cr_20_Mn_20_Fe_20_Co_20_Ni_20_ are more anisotropic than hcp Cr_20_Mn_20_Fe_30_Co_20_Ni_10_ and fcc Cr_10_Mn_30_Fe_50_Co_10_, respectively.

**Table 3:** Young’s modulus *E* in several high-symmetry directions, the anisotropy factor of Young’s modulus *f*_*E*_, and the elastic anisotropy *A*_*u*_ of HEAs in the hcp and fcc phases.

HEA	fcc					hcp			
	*E*_[001]_	*E*_[110]_	*E*_[111]_	${{\boldsymbol{f}}}_{{\boldsymbol{E}}}^{{\bf{fcc}}}$	*A*_*u*_	${{\boldsymbol{E}}}_{{\bf{[2}}\bar{{\bf{1}}}\bar{{\bf{1}}}{\bf{0}}{\boldsymbol{]}}}$	*E*_[0001]_	${{\boldsymbol{f}}}_{{\boldsymbol{E}}}^{{\bf{hcp}}}$	*A*_*u*_
Cr_10_Mn_30_Fe_50_Co_10_	162.7	310.1	444.3	3.40	2.03	379.1	523.9	1.38	0.17
Cr_20_Mn_20_Fe_34_Co_20_Ni_6_	141.3	283.2	425.8	3.70	2.36	331.5	458.9	1.38	0.22
Cr_20_Mn_20_Fe_30_Co_20_Ni_10_	136.9	276.3	418.2	3.75	2.42	321.7	442.6	1.37	0.23
Cr_20_Mn_20_Fe_20_Co_20_Ni_20_	128.5	262.5	402.6	3.85	3.12	284.8	404.6	1.42	0.29
Cr_25_Fe_25_Co_25_Ni_25_	138.7	275.6	411.0	3.80	2.48	248.3	379.8	1.53	0.39

**Figure 2:**
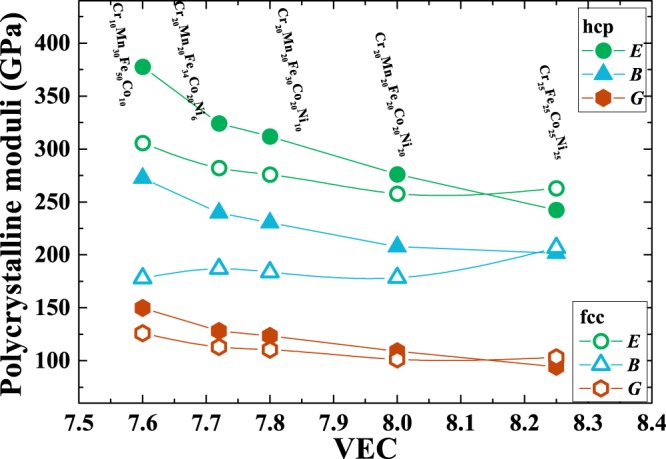
Polycrystalline moduli of the HEAs in the hcp and fcc phases as a function of their VEC.

We derived the polycrystalline Young’s modulus *E*, bulk modulus *B*, and shear modulus *G* employing the calculated single-crystal elastic constants, which were determined using the Voigt-Reuss-Hill average method^31^. Fig. 2 displays the polycrystalline moduli as a function of average valance electron concentration (VEC). From Fig. 2, we can see that all the moduli of the five HEAs in the hcp phase decrease with increasing VEC, and the variations are $\sim \mathrm{38 \% }$ for *G*, $\sim \mathrm{36 \% }$ for *E*, and $\sim \mathrm{26 \% }$ for *B*. Turning to the fcc phase, it can be seen that *E* and *G* reduce until the VEC reaches ≈ 8, then both slightly increase. We find that the decreases are about 20% and 16% for *G* and *E*, respectively. Nevertheless, for *B*, the variation is very small when the VEC is lower than 8, but it exhibits a large increase when the VEC approaches 8.25. Comparing these two phases, we observe that all presented HEAs in their hcp phase have larger *E*, *B*, *G* than in their fcc phase when the VEC ≤ 8. Interestingly, fcc Cr_25_Fe_25_Co_25_Ni_25_ with a VEC of 8.25 has larger moduli compared with its hcp phase.

To get further insight into the elastic anisotropy in these HEAs, we compare the fcc and hcp phases. To this end, we employ a universal elastic anisotropy parameter *A*_*u*_. Different from other anisotropy indicators, such as the commonly used Zener anisotropy ratio^32^, *A*_*u*_ allows direct comparison of the anisotropy of different crystal structures and is defined as^33^.

3 $${A}_{u}=5\frac{{G}_{V}}{{G}_{R}}+\frac{{B}_{V}}{{B}_{R}}-6.$$

Here, the indexes *V* and *R* denote averaging through the Voigt and Reuss methods, respectively. It should be noted that ${G}_{V}\ge {G}_{R}$ and ${B}_{V}\ge {B}_{R}$^31,34^, hence ${A}_{u}\ge 0$. For an isotropic crystal, *A*_*u*_ is zero, and its deviation from zero gives a measure of the anisotropy.

The derived values are summarized in Table 3. For the fcc phase, we observe that Cr_10_Mn_30_Fe_50_Co_10_ has the smallest *A*_*u*_, whereas Cr_20_Mn_20_Fe_20_Co_20_Ni_20_ has the largest one. This trend reflects that of ${f}_{E}^{{\rm{fcc}}}$. For the hcp phase, *A*_*u*_ is close to zero and increases with increasing VEC. Comparing the two close-packed phases, we conclude that the fcc structure is elastically more anisotropic than the hcp structure. This result confirms two previous findings for elements and binary alloys, namely that for the same material, the anisotropy of the fcc phase exceeds that of the hcp phase^35^, and the hexagonal crystal class is the class with least *A*_*u*_^33^.

### Ideal shear strength

**Figure 3:**
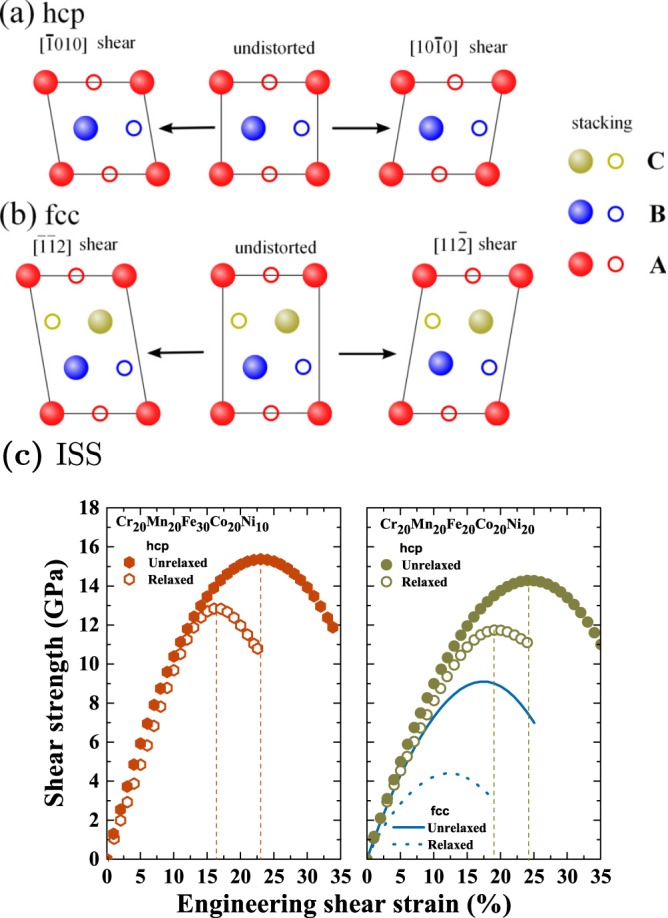
(**a**) Schematics of the $$\mathrm{(0001)[10}\bar{1}\mathrm{0]}$$ affine shear deformation of the hcp structure viewed along the $$\mathrm{[11}\bar{2}\mathrm{0]}$$ direction. The sheared conventional unit cell is monoclinic and contains four atoms. The $$\mathrm{[10}\bar{1}\mathrm{0]}$$ and $$[\bar{1}\mathrm{010]}$$ shearing directions are equivalent. (**b**) Schematics of the (111)[11 $$\bar{{\rm{2}}}$$] affine shear deformation of the fcc structure viewed along the [1 $$\bar{{\rm{1}}}$$ 0] direction. The [11 $$\bar{{\rm{2}}}$$] and [$$\bar{{\rm{1}}}$$$$\bar{{\rm{1}}}$$ 2] shearing directions are not equivalent ([11 $$\bar{{\rm{2}}}$$] is the soft direction, [$$\bar{{\rm{1}}}$$$$\bar{{\rm{1}}}$$ 2] the hard one). In (a) and (**b**) the undistorted cells coincide with the orthorhombic representation of the hcp and the fcc structures, respectively. A, B, and C denote the stacking sequence, and filled and open circles distinguish atoms in the two types of $$\mathrm{(11}\bar{2}\mathrm{0)}$$ and (1 $$\bar{{\rm{1}}}$$ 0) planes (these crystal planes lie in the figure plane). (**c**) The shear stress of hcp Cr_20_Mn_20_Fe_30_Co_20_Ni_10_ and Cr_20_Mn_20_Fe_20_Co_20_Ni_20_ under $$\mathrm{(0001)[10}\bar{1}\mathrm{0]}$$ shear deformation as a function of applied shear strain [corresponding to (**a**)]. The fcc data were taken from ref.^43^.

The elastic constants and polycrystaline moduli considered so far describe the mechanical properties of materials in the small deformation region, where the stress-strain relations are linear. In the following, we go beyond the linear elasticity regime and consider the resistance of the ideal fcc and hcp single crystals against shear within the (111) and (0001) planes, respectively. The ideal strength is the applied stress at which a perfect crystal becomes mechanically unstable. It is an intrinsic property of a material and can provide insight into the correlation between chemical bonding and crystal symmetry. The ideal strength has been accepted as a mechanical parameter for the design of high performance materials^36–38^. It has been demonstrated that the measured strengths approach the strengths obtained from first-principles electronic structure calculations in some cases^38–42^, e.g., for whiskers. In this work, we selected two HEAs in the hcp structure, i.e., Cr_20_Mn_20_Fe_30_Co_20_Ni_10_ and Cr_20_Mn_20_Fe_20_Co_20_Ni_20_, and calculated their ideal shear strengths (ISSs) on the basal plane [(0001)] along the $\mathrm{[10}\bar{1}\mathrm{0]}$ direction. For Cr_20_Mn_20_Fe_20_Co_20_Ni_20_ in the fcc phase, the ISS on the (111) plane along the [11 $\bar{{\rm{2}}}$] direction was reported previously^43^. It should be noted that the chosen shear systems in the fcc and hcp structures are equivalent, see Fig. 3 and discussion below. A monoclinic computational cell was employed to model the affine shear deformation, which is displayed in Fig. 3(a). The shear stress *τ*(*γ*) is given by

4 $$\tau (\gamma )=\frac{1}{{\rm{\Omega }}(\gamma )}\frac{\partial {\rm{E}}}{\partial \gamma },$$

where *E* is strain energy per atom and Ω(*γ*) is the volume at each shear strain *γ* defined as the ratio of displacement along the $\mathrm{[10}\bar{1}\mathrm{0]}$ direction to the height of the unit cell. The first maximum on the stress-strain curve Eq. (4) determines the ISS *τ*_m_ with corresponding engineering maximum shear strain *γ*_m_. Here, two modes of shearing were considered^44^: (i) no relaxation is allowed after shearing; (ii) relaxation is allowed after each shear step, the only constraint being the shearing angle. (The underlying primitive unit cell governing the affine shear deformation contains one atom for the fcc phase and two atoms for the hcp phase. Relaxation involved volume and shape of the cell but a possible phonon instability was not investigated.)

Figure 3(c) presents the obtained stress-strain curves for Cr_20_Mn_20_Fe_30_Co_20_Ni_10_ and Cr_20_Mn_20_Fe_20_Co_20_Ni_20_. For the relaxed case, we found that the ISSs are 12.8 GPa and 11.7 GPa for Cr_20_Mn_20_Fe_30_Co_20_Ni_10_ and Cr_20_Mn_20_Fe_20_Co_20_Ni_20_, respectively. Compared to the relaxed ISSs, we realize that the ISSs neglecting lattice relaxation during shearing are 22% and 20% larger for Cr_20_Mn_20_Fe_30_Co_20_Ni_10_ and Cr_20_Mn_20_Fe_20_Co_20_Ni_20_, respectively. It is noteworthy that the obtained ISS of Cr_20_Mn_20_Fe_20_Co_20_Ni_20_ in the hcp phase is more than two times larger than that in the fcc phase (4.4 GPa)^43^, see Fig. 3(c). Moreover, compared to the fcc phase, the relaxation effect on the ISSs is less pronounced in the hcp phase, see Fig. 3(c).

The differences in the ISS can be explained as follows. Upon affine shear deformation, atoms in a close-packed layer gradually move over those in subjacent layers; see Fig. 3(a) and (b). For both the hcp and fcc structures this process involves atoms in two types of $\mathrm{(11}\bar{2}\mathrm{0)}$ and $\mathrm{(1}\bar{1}\mathrm{0)}$ planes, respectively. An atom moving over another atom (in adjacent close-packed layers) leads to a steeper increase in energy when they are located in the *same* type of $\mathrm{(11}\bar{2}\mathrm{0)}$ or $\mathrm{(1}\bar{1}\mathrm{0)}$ plane than when they are located in the *other* type of $\mathrm{(11}\bar{2}\mathrm{0)}$ or $\mathrm{(1}\bar{1}\mathrm{0)}$ plane. This is plausible since in the former case the two atoms directly move over one another, while in the latter case they are shifted by 1/2 $\mathrm{[11}\bar{2}\mathrm{0]}$ or 1/2 $\mathrm{[1}\bar{1}\mathrm{0]}$. In the case of shearing the hcp unit cell, an equal number of atoms moves over atoms located in the same type of $\mathrm{(11}\bar{2}\mathrm{0)}$ plane and located in the other type of $\mathrm{(11}\bar{2}\mathrm{0)}$ plane. This atomic displacement is independent of the shearing direction, i.e., $\mathrm{[10}\bar{1}\mathrm{0]}$ and $[\bar{1}\mathrm{010]}$ are equivalent; see Fig. 3(a). When shearing the fcc structure in the $\mathrm{[11}\bar{2}]$ direction, all atoms located in one type of $\mathrm{(1}\bar{1}\mathrm{0)}$ plane move over atoms located in the other type of $\mathrm{(1}\bar{1}\mathrm{0)}$ plane, whereas for shear in the opposite, $[\bar{1}\bar{1}\mathrm{2]}$ direction all atoms located in one type of $\mathrm{(1}\bar{1}\mathrm{0)}$ plane move over atoms located in the same type of $\mathrm{(1}\bar{1}\mathrm{0)}$ plane. This leads to a steeper increase in energy and larger stress for shear in the $[\bar{1}\bar{1}\mathrm{2]}$ direction (hard direction, ${\tau }_{m}^{{\rm{hard}}}$) than for shear in the $\mathrm{[11}\bar{2}]$ direction (soft direction, ${\tau }_{m}^{{\rm{soft}}}$). Due to the ABAB stacking sequence, the shearing of the hcp structure may be viewed as being the intermediate case between these two limits. Thus, it is expected that the energy raises more rapidly (less rapidly) as a function of shear strain than in the soft (hard) direction of the fcc structure and the maximum shear stress is approximately given by the arithmetic average, $({\tau }_{m}^{{\rm{hard}}}+{\tau }_{m}^{{\rm{soft}}}\mathrm{)/2}$. A previous DFT study for fcc Ni showed that ${\tau }_{m}^{{\rm{hard}}}\approx 3{\tau }_{m}^{{\rm{soft}}}$^45^.

### Half-width of the dislocation core

It has been recognized that the half-width of the dislocation core *ζ* plays an important role in the prediction of the Peierls stress^46^. The half-width of the dislocation core *ζ* may be derived using the calculated ISS based on the following equation^46^.

5 $$\zeta =\frac{Kb}{4\pi {\tau }_{m}},$$

where *b* is the magnitude of the Burgers vector, and *K* is the energy factor of the dislocation. Here, we consider an edge dislocation in the basal plane (0001) with dislocation line along the $\mathrm{[10}\bar{1}\mathrm{0]}$ direction and Shockley partial Burgers vector ${\bf{b}}=\frac{1}{3}\mathrm{[10}\bar{1}\mathrm{0]}$. The corresponding energy factor *K* can be calculated viz^47^,

6 $$K=(C+{C}_{13})[\frac{{C}_{44}(C-{C}_{13})}{{C}_{33}(C+{C}_{13}+2{C}_{44})}],$$

with

7 $$C={({C}_{11}{C}_{33})}^{\mathrm{1/2}}.$$

The derived *ζ* are 1.57 Å and 1.53 Å for Cr_20_Mn_20_Fe_30_Co_20_Ni_10_ and Cr_20_Mn_20_Fe_20_Co_20_Ni_20_, respectively. It is found that the values of *ζ* for both HEAs are very close to their corresponding Burger vectors, i.e., 1.43 Å for Cr_20_Mn_20_Fe_30_Co_20_Ni_10_ and 1.44 Å for Cr_20_Mn_20_Fe_20_Co_20_Ni_20_. For fcc Cr_20_Mn_20_Fe_20_Co_20_Ni_20_, *ζ* of an edge dislocation having a (111) glide plane with dislocation line along the $\mathrm{[1}\bar{1}\mathrm{0]}$ direction was calculated previously^43^. It should be noted that the chosen edge dislocation configurations in the fcc and hcp close-packed planes are equivalent. Compared with the *ζ* of the hcp phase, the *ζ* of the fcc phase (3.89 Å) turns out to be more than two times larger. According to the Peierls-Nabarro model, a wider dislocation core combined with a similar Burgers vector leads to a reduced Peierls barrier^48^. This result indicates that the fcc phase is more ductile than the hcp one.

## Conclusion

In summary, a detailed first-principles investigation of the mico-mechanical properties of five polymorphic HEAs alloys in their fcc and hcp phases was presented. From the obtained elastic constants, it was found that all considered HEAs are elastically stable in both phases. We found that the polycrystalline moduli *B*, *G*, and *E* of the considered HEAs in the hcp phase decrease with increasing the VEC. The biggest and smallest variations were noticed in *G* and *B*, respectively. Turning to the fcc phase, we observed that *E*, *G*, and *B* show a non-monotonic behavior as a function of VEC. Comparing these two phases, we noticed that all presented HEAs in the hcp phase have larger *E*, *B*, and *G* when the VEC is below 8. We observed that the investigated HEAs are elastically more anisotropic in the fcc phase than in the hcp phase. We found that the obtained ISS for the selected Cr_20_Mn_20_Fe_20_Co_20_Ni_20_ in its hcp phase is two times larger than in its fcc phase. In addition, the relaxation effect on the ISSs of the hcp phase was found to be less pronounced than that of the fcc phase. The calculated half-widths of the dislocation core for Cr_20_Mn_20_Fe_20_Co_20_Ni_20_ suggested that the Peierls barrier in the hcp phase is larger than in the fcc phase. The present results are expected to offer a guideline for further developing high performance HEAs.

## Methods

The first-principles method used in this study is based on density-functional theory (DFT)^49^, and the Kohn-Sham equations were solved using the exact muffin-tin orbitals method (EMTO)^50–52^. For the self-consistent determination of the charge density and the total energy calculations, we employed the Perdew-Burke-Ernzerhof functional^53^. Here, all calculations were performed in the paramagnetic state, which was described by the disordered-local moment model^54^. The problem of chemical disorder was treated within the coherent-potential approximation (CPA) and the total energy is computed via the full charge-density technique^55,56^. The basis set included s, p, d, and f states. Brillouin zone integrations were performed on a 33 × 33 × 33 and a 31 × 31 × 25 k-points mesh for the fcc and hcp single-crystal elastic constants calculations, respectively. In this work, for the equation of state calculations, we used the primitive cell for the fcc and hcp phases. For the elastic constants calculations, the unit cell with highest symmetry compatible with the prescribed deformation (including the unstrained state) were employed; for further methodological details, see ref.^56^. Based on the accuracy of our numerical fits to the computed energy versus strain curves and on the Brillouin zone sampling, all elastic parameters and ISSs are estimated to posses error bars below 1 GPa and 0.5 GPa, respectively. The accuracy of the EMTO-CPA method for the equation of state, elastic properties, and ideal strength of alloys has been demonstrated in a number of previous works^57–59^.
